# Your Space or Mine? Mapping Self in Time

**DOI:** 10.1371/journal.pone.0049228

**Published:** 2012-11-15

**Authors:** Brittany M. Christian, Lynden K. Miles, C. Neil Macrae

**Affiliations:** School of Psychology, University of Aberdeen, Aberdeen, United Kingdom; McMaster University, Canada

## Abstract

While humans are capable of mentally transcending the here and now, this faculty for mental time travel (MTT) is dependent upon an underlying cognitive representation of time. To this end, linguistic, cognitive and behavioral evidence has revealed that people understand abstract temporal constructs by mapping them to concrete spatial domains (e.g. past = backward, future = forward). However, very little research has investigated factors that may determine the topographical characteristics of these spatiotemporal maps. Guided by the imperative role of episodic content for retrospective and prospective thought (i.e., MTT), here we explored the possibility that the spatialization of time is influenced by the amount of episodic detail a temporal unit contains. In two experiments, participants mapped temporal events along mediolateral (Experiment 1) and anterioposterior (Experiment 2) spatial planes. Importantly, the temporal units varied in self-relevance as they pertained to temporally proximal or distal events in the participant’s own life, the life of a best friend or the life of an unfamiliar other. Converging evidence from both experiments revealed that the amount of space used to represent time varied as a function of target (self, best friend or unfamiliar other) and temporal distance. Specifically, self-time was represented as occupying more space than time pertaining to other targets, but only for temporally proximal events. These results demonstrate the malleability of space-time mapping and suggest that there is a self-specific conceptualization of time that may influence MTT as well as other temporally relevant cognitive phenomena.

## Introduction

“Space by itself and time by itself are doomed to fade away into mere shadows, and only a kind of union of the two will preserve an independent reality.”

Albert Einstein

While time travel remains an entertaining impossibility, the mind at least is unconstrained by physical laws that ground the body in the present. By mentally replaying past episodes and foreseeing future events, people’s subjective experiences routinely transcend the here-and-now [1]–[5]. This psychological ability to traverse time rests on a couple of basic requirements: (i) an underlying cognitive representation of time [5]; and (ii) a neuro-anatomical network that supports temporal self-projection [6]. Interestingly, while an extensive literature has identified regions of the brain that support mental time travel (MTT), [2], [7]–[10] considerably less is known about the structural properties of temporal representation. The problem is absorbing, how do people characterize something as intangible as time? As it turns out, to resolve the puzzle of temporal construal the mind employs a clever strategy – abstract temporal concepts (e.g., *past*, *future*) are translated into concrete spatial representations, a tactic that is consistent with theories of magnitude [11], metaphoric cognition [12]–[15] and embodiment [16]. Put simply, people use space to think about time.

Pervading language, cognition and action, examples of space-time mapping abound. For example, people talk of putting the past *behind* them and focusing on the year *ahead* [17]. Beyond the application of such linguistic metaphors, gestural patterns, movement dynamics and attentional processing also reveal that temporal information is systematically prescribed to spatial locations [18]–[25]. Notwithstanding the universal occurrence of this psychological phenomenon [14], [15] the manifold characteristics of space-time mapping are subject to important cultural variation. Notably, precisely where temporal concepts are located in space (e.g., forward, back, left, right, up, down) is flexible [25] and can be impacted both by sociolinguistic custom (e.g., reading/writing direction) [22], [26]–[30] and experiential factors (e.g., movement of the sun) [31].

The fact that sociolinguistic convention (e.g., reading/writing direction) provides a plane onto which time can be mapped gives rise to some important effects. Along this culturally defined axis, time is portrayed as a linear progression of events (i.e., the flow of time) with the present moment lying at the intersection of that which has already happened and experiences which have yet to occur [32]. Taking the form of a mental timeline (MTL) [33] this representation is used to spatially organize events of personal, cultural and historical significance. Evidence corroborating the existence of a MTL comes from the spatial-temporal association of response codes or STARC effect [34]. In a seminal investigation, it was shown that when asked to associate sequential daily events (e.g., breakfast, lunch, dinner) with locations in space, American children (left-to-right reading/writing direction) followed the early-left/late-right ordering of events, whereas Arab children (right-to-left reading/writing direction) displayed the opposite pattern [30]. Replicating and extending these findings to chronometric measures, recent work has revealed a bias in the ease with which manual responses can be elicited by stimuli with temporal connotations. For example, while past-related words are responded to most quickly with the left hand, future-related words yield a right-hand advantage – an effect that is reversed in Hebrew speakers [35] (for related findings see [19], [27], [28], [36]).

While cultural forces exert a significant influence on the directionality of mental timelines (e.g., horizontal vs. vertical) [27] other factors that reliably impact the spatialization of time have yet to be elucidated. In this respect, the nature of recollective experiences (i.e., event simulation) may play a pivotal role in shaping the structural characteristics of space-time mapping. It is well established that episodic memories serve as the building blocks of MTT [4], [9]. Both neuropsychological and behavioral investigations converge on two important findings. First, simulating future outcomes on the basis of prior experience relies on overlapping neural structures and cognitive operations [2], [8], [37]. In other words, there is an explicit connection between episodic memory and episodic-future thought [2], [9]. Second, impairments in episodic memory impede the efficacy of MTT [4], [7], [38]–[40]. Specifically, difficulties in remembering events from the past translate into problems envisaging episodes in the future. Therefore, if episodic content provides a basis for MTT which, in turn, is supported by spatial representations of time, might the nature of recollective and prospective experience also impact the topography of space-time mapping?

Aside from brain damage, illness, and aging [9] a critical determinant of the characteristics (e.g., complexity, detail, richness) of episodic memory is the target to which the recollections apply. According to the self-reference effect (SRE), people remember more information about themselves than any other individual [41]–[44]. Moreover, the amount and quality of episodic detail retained for others diminishes as targets become less familiar [45]. Thus, one is likely to remember very little about the dentist, a great deal about Uncle Frank and an enormous amount about self. These differences in episodic memory raise an interesting possibility. Perhaps the spatial representation of time is sensitive to the amount of self-relevant episodic detail a given temporal period contains. Specifically, the spatial extent of time (i.e., how much space a unit of time occupies) may reflect the richness of episodic content (e.g., more detail = more time/space) [11].

If operating, such a space-time mapping effect gives rise to noteworthy predictions. First, self-time should occupy more space than a comparable temporal period for any other target (e.g., best friend). Second, this spatialization effect should be more pronounced for temporally proximal (i.e. near) than distal (i.e. far) periods, as both recollection and future-based simulation are known to decrease dramatically in both frequency and detail as a function of increasing temporal distance from the present [46]–[54]. Of note, this latter prediction is consistent with Trope and Liberman’s influential construal level theory of psychological distance [55]–[57]. As psychological distance (spatial, temporal, social) increases, mental construal is routinely characterized by a shift from concrete (e.g., episodic) to abstract (e.g., semantic) representations [57]–[60]. What this again suggests is that target-based effects on the spatialization of time should be more evident for proximal than distal temporal eras. We explored these predictions using both spatial (Experiment 1) and temporal (Experiment 2) measures of space-time mapping.

## Experiment 1

The purpose of Experiment 1 was to provide an initial investigation into the effects of target familiarity and temporal proximity on the spatial representation of time. Drawing from related work regarding the assessment of mediolateral (i.e., left-to-right) number-space mappings [61], we adapted a simple line segmentation task whereby participants located events (i.e., birthdays) along a horizontal line. More specifically, participants marked the location of either their own, their best friend’s or a hypothetical stranger’s birthdays in the past (i.e., 8^th^ and 9^th^ birthdays), present (i.e., previous and next birthdays) and future (i.e., 58^th^ and 59^th^ birthdays). In this way, participants were in fact denoting the spatial boundaries of a fixed unit of time (i.e., one year). If differences in episodic content influence the mapping of time to space, then reductions in such detail (i.e., via diminished self-relevance or increased temporal remoteness) should be accompanied by decreases in the extent to which a single year is represented spatially.

## Methods

### 

#### Ethics statement

The study was reviewed and approved by the School of Psychology, University of Aberdeen Ethics Committee. All participants gave written informed consent prior to taking part.

#### Participants and design

Sixty participants (42 female), aged between 18 and 32 years (*M = *22.6 years) from the University of Aberdeen took part in an experiment exploring the mental representation of time. A 3 (Target: self, best friend, unfamiliar other)×3 (Time: past, present, or future) mixed-model design with repeated measures on the second factor was employed.

#### Procedure and materials

The experiment employed a line segmentation task (see [61] for a similar method) in which participants were asked to indicate the location of events along a hypothetical timeline. Participants were presented with a horizontal black line (360 mm) printed on a standard A3 (297×430 mm) sheet of white paper. The line was unbounded [62] and bisected with a vertical line (10 mm), which was labeled ‘NOW’. Labeling the line oriented the participants to a temporal framework and attempted to dispel any assumptions that the line was a “life line” (i.e., beginning at birth and ending at death) as the age of our participants would not suggest that they were currently half way through their lives.

Participants were initially given instructions regarding the target whose birthdays they would be locating on the timeline (*n* = 20 per condition). Those in the ‘self’ condition were instructed to mark their own birthdays while those in the ‘best friend’ condition were instructed to think of a close friend similar in age to themselves. Finally, participants in the ‘unfamiliar other’ condition were told to think of a hypothetical stranger whose birth date was the same as theirs. Next, participants were asked to mark the location on the timeline of six specific birthdays, two representing the past (i.e., 8^th^ and 9^th^ birthdays), two representing the present (i.e., previous and next birthdays) and two representing the future (i.e., 58^th^ and 59^th^ birthdays). The experimenter presented the birthdays one at a time (i.e., allowing for a response to be made before continuing to the next) in a unique random order for each participant. After completing all six trials participants were debriefed and dismissed.

## Results and Discussion

All participants followed a left-to-right ordering of time whereby birthdays from the past were located to the left side of the line, while those in the future were marked on the right side. The size of participants’ spatial representations of one year in time was assessed by measuring (in mm) the distance between the marks corresponding to the two birthdays representing the past (i.e., 8^th^ and 9^th^), present (i.e., previous and next) and future (i.e., 58^th^ and 59^th^) periods separately. These distances were compared using a 3 (Target: self, best friend, unfamiliar other) ×3 (Time: past, present, future) mixed-model analysis of variance (ANOVA), with repeated measures on the second factor. This revealed main effects of Target, *F*(2, 57) = 6.42, *p* = .003, *η*_p_^2^ = 0.18, whereby the amount of space used to represent one year increased as a function of target familiarity (i.e., self > best friend > unfamiliar other; Tukey *a*, *p*<.05); and Time, *F*(2,114) = 43.64, *p*<.001, *η*_p_^2^ = 0.43, whereby more space was used to represent one year in the present than in the past or the future (i.e., present >past = future; Tukey *a*, *p*<.05).

**Figure 1:**
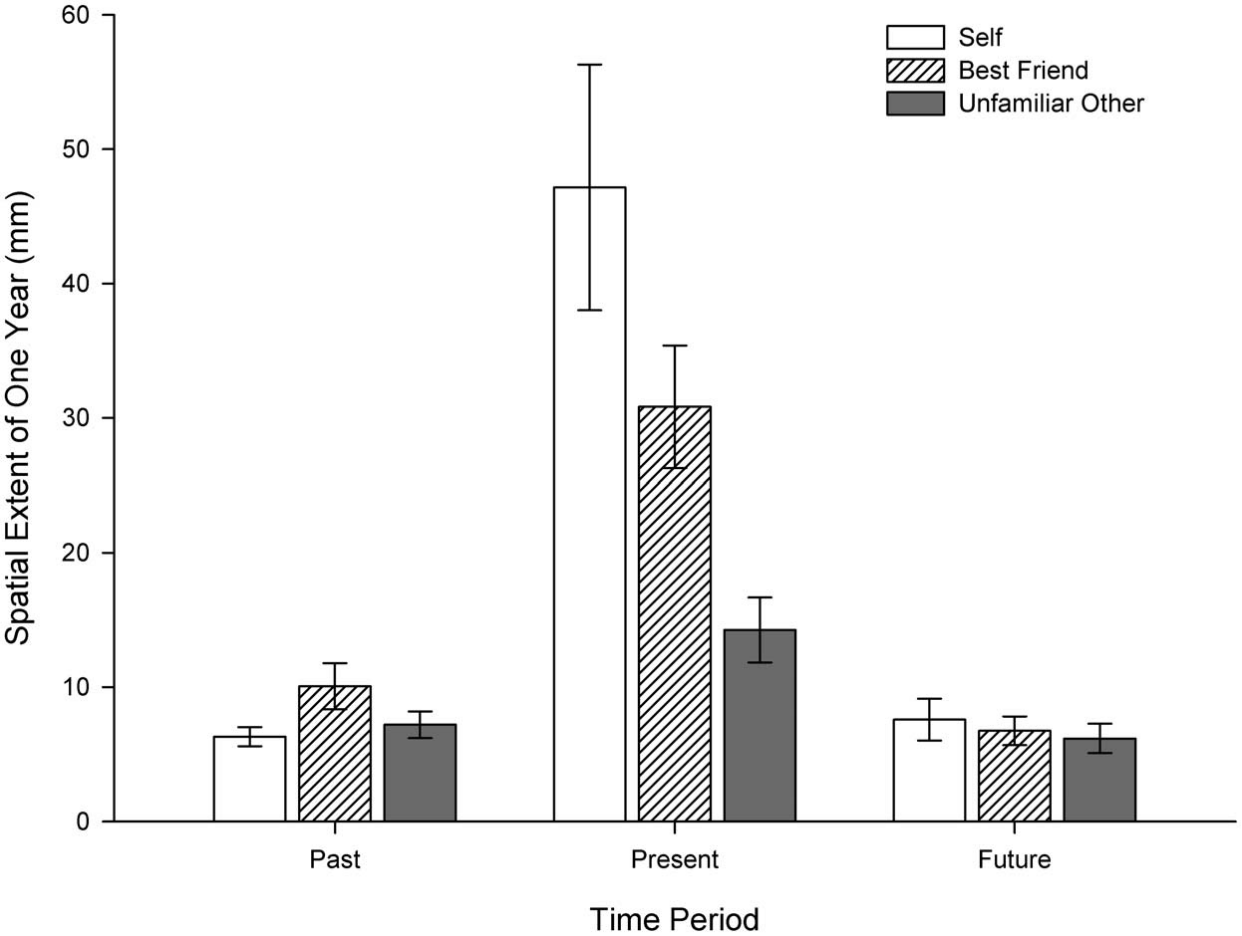
Mediolateral Spatialization of Time. Spatial representation of one year (mm) as a function of time period (i.e., past, present, future) and target (i.e., self, best friend, unfamiliar other) in Experiment 1. Error bars represent ±1SEM.

Importantly, these effects were qualified by a Target×Time interaction, *F*(4, 114) = 2.82, *p*<.001, *η*_p_^2^ = .20 (see Figure 1). To examine this effect further, simple main effects analyses were performed to compare the effect of target at each time period. Although no differences were found as a function of target for either the past or future periods, a significant effect was revealed for the present time period, *F*(2, 57) = 7.37, *p* = .001. Of note, there was a strong linear trend (*p*<.001) such that participants in the ‘self’ condition used the most space to represent one year in the present (*M = *47.2 mm), followed by participants in the ‘best friend’ condition (*M = *30.8 mm) and finally those in the ‘unfamiliar other’ condition (*M = *14.3 mm).

These results suggest that both the target and the time period influenced the amount of space participants used to represent one year in time. Where the amount of episodic content might be expected to be greater (i.e., for self-relevant and temporally proximal contexts), one year of time occupied more space than for less episodically rich events. In particular, when considering the present year, a strong positive linear relationship was observed between target familiarity and the extent of the spatial representation of time. That is, consistent with the predicted effects, a year relative to self occupied more space than the same time period relative to a close friend, which in turn was represented as larger than a year in the life of an unfamiliar other. Interestingly, although a year in the present was consistently represented as larger than a year in either the past or future, no difference was found between the latter two time periods despite an asymmetry in their respective temporal distances from the present (i.e., for all the participants their 8^th^ and 9^th^ birthdays were more temporally proximal than their 58^th^ and 59^th^ birthdays). One possibility here is that the remoteness in time of these events relegate them to be represented as generically in the past/future without reference to specifically how temporally distant they might be. If this is the case, the lack of target effects at the past and future time points may be due to relatively less concrete representations of time when temporally remote events are considered [55]. We sought to replicate and extend these findings in Experiment 2 by focusing on a more constrained time-frame (i.e., ±10 years), and employing an alternative, temporally-based index of space-time mapping.

## Experiment 2

Experiment 2 was designed to conceptually replicate the relationship between episodic content and the scaling of spatiotemporal mappings reported above. Importantly, a novel method was employed that required participants to estimate the duration of a hypothetical journey to specific events (i.e., birthdays) in the past and future. This approach had two substantive points of difference from the method used in Experiment 1. First, participants mapped time to space along the anterioposterior (i.e., front-to-back) plane whereby, at least amongst English speakers, the past metaphorically lies behind and the future in front [17]. Second, participants engaged in a more dynamic task in which they were exposed to patterns of optic flow designed to simulate self-motion while they ‘travelled’ to target events. Such displays reliably induce experiences of vection (i.e., apparent self-motion) [63], shape the temporal locus of MTT [20], and support mental simulations of long distance travel [64]. In this latter study, participants were asked to imagine travelling to (spatially) distant locations with the duration of exposure to the optic flow display used to measure the length of the journeys. The current experiment adapted this technique, asking participants to ‘time travel’ to their own or others’ birthdays in the past and future while viewing centripetal or centrifugal optic flow patterns (i.e., specifying backwards and forwards movement respectively).

## Method

### 

#### Ethics statement

The study was reviewed and approved by the School of Psychology, University of Aberdeen Ethics Committee. All participants gave written informed consent prior to taking part.

#### Participants and design

Sixty-three participants (37 female), aged between 18 and 32 years (*M* = 21.6 years) from the University of Aberdeen took part in an experiment exploring the perception of time. A 3 (Target: self, best friend, unfamiliar other) ×2 (Temporal direction: past, future) ×10 (Temporal distance: 1–10 years from present) mixed-model design with repeated measures on the final two factors was employed.

#### Procedure and materials

**Figure 2:**
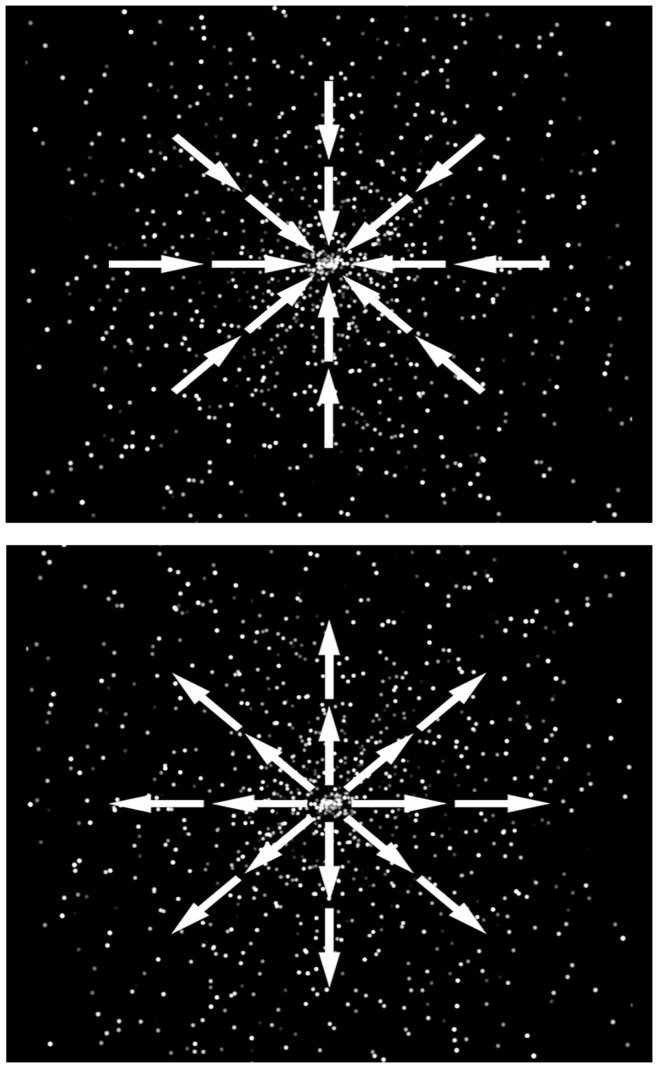
Vection Displays. Illustrations of the direction of optical flow specified by the star-field displays in Experiment 2. The top panel shows centripetal flow specifying backwards vection (i.e., past trials) while the bottom panel shows centrifugal flow specifying forwards vection (i.e., future trials).

A dynamic star-field display adapted from previous work [20] was employed to induce vection (i.e., apparent self motion) and support the experience of ‘travelling’ to the target events. The display consisted of approximately 1000 white dots (i.e., stars) animated (25 fps) so as to move either toward (i.e., centripetally) or away from (i.e., centrifugally) the centre of the display (see Figure 2), corresponding to the experience of backward and forward self motion respectively [63].

Upon arrival participants were told that their task was to operate a notional ‘time machine’ which they would be required to stop at various events in the past or future. They were seated at a desk approximately 2.5 m away from a large screen onto which the animated star-field display was projected (image size: 1.45×1.10 m). A response box with a green and a red button was used by participants to begin (i.e., initiate the star-field animation) and end (i.e., stop the animation) each trial (i.e., ‘time travel’ event). Initially participants completed two practice trials, one accompanied by the centripetal (i.e., backward) and the other by the centrifugal (i.e., forward) star-field display, in order to familiarize them with the procedure.

Next participants were given instructions regarding the events to which they would be travelling (*n* = 21 per condition). In line with Experiment 1, those in the ‘self’ condition travelled to their own birthdays, those in the ‘best friend’ condition travelled to the birthdays of a close friend similar in age to themselves, while those in the ‘unfamiliar other’ condition travelled to the birthdays of a hypothetical stranger whose birth date was the same as theirs. Participants were then asked their age, which was used to calculate the target birthdays (e.g., a 20 year old participant travelling 5 years into the future would be asked to stop the ‘time machine’ at their 25^th^ birthday). Each trial began with a target destination presented on the screen (e.g., Please travel to your friend’s 14^th^ birthday) and participants then started the ‘time machine’ (i.e., the star-field display), stopping it again once they felt they had arrived at the target birthday. Target destinations in the past were accompanied by the centripetal (i.e., backward) star-field display while those in the future were accompanied by the centrifugal (i.e., forward) display. Each participant completed 20 trials (1–10 years in the past and future) in a unique random order. Eprime 2.0 was used to present the trials and record the length of time participants took to ‘travel’ to each birthday (i.e., the time elapsed between starting and stopping the ‘time machine’). Because the star-field animations were always presented at a constant speed, the duration of each ‘journey’ corresponded to the distance travelled, that is, the amount of space used to represent a given period of time.

## Results and Discussion

In contrast to Experiment 1 where discrete units of time were the focus of the analyses, here we treated time as a continuous construct. That is, instead of comparing individual 1-year units of time we investigated more general patterns by initially fitting each participant’s data with a regression line for the past and future trials separately. This yielded parameter estimates for the y-intercept, corresponding to the ‘size’ of the hypothetical present (i.e., time ±0), and slope, corresponding to the change in the ‘size’ of time as a function of temporal distance from the present (i.e., time ±1–10 years), of each line.

**Figure 3:**
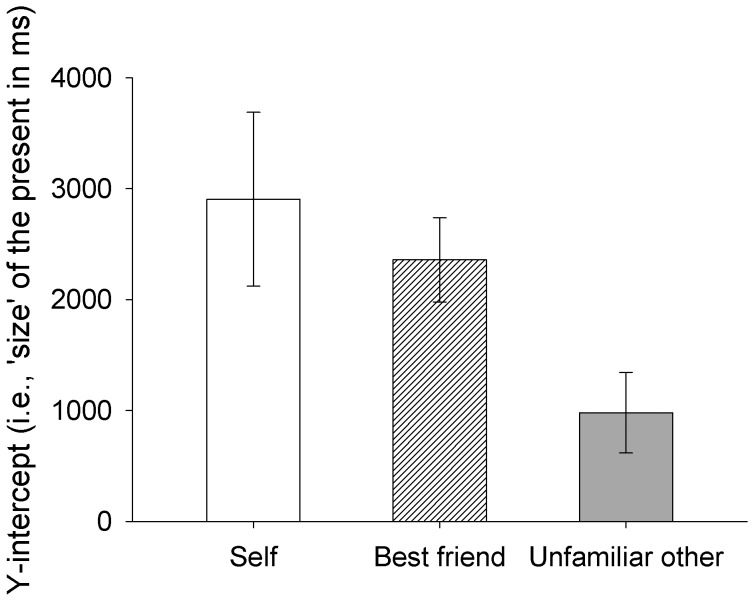
Anterioposterior Spatialization of Time. Temporal representation of the ‘size’ of the hypothetical present (i.e., y-intercept representing time ±0) as a function of target (i.e., self, best friend, unfamiliar other) in Experiment 2. Error bars represent ±1SEM.

The y-intercepts of the regression lines were compared using a 3 (Target: self, best friend, unfamiliar other) ×2 (Temporal direction: past, future) mixed-model ANOVA with repeated measures on the second factor. This revealed a main effect of Target, *F*(2,60) = 3.32, *p* = .04, η_p_^2^ = 0.10 (see Figure 3). Follow-up analyses comparing the three Target conditions again revealed evidence of a strong linear trend (*p* = .015) whereby participants travelled farther (i.e., used more space) to events that were more self-relevant (i.e., self > best friend > unfamiliar other). There was no effect of Temporal direction, suggesting symmetrical spatial representations of past and future events, nor was there a Target×Temporal direction interaction.

The slopes of the regression lines were compared using a 3 (Target: self, best friend, unfamiliar other) ×2 (Temporal direction: past, future) mixed-model ANOVA with repeated measures on the second factor. No significant effects were revealed. Inspection of the overall mean slope indicates a general positive relationship between time and space. Specifically, on average participants took 836 ms to travel each additional year in time.

Taken together, these results provide further evidence that variations in self-relevant episodic content relative to past and future events are reflected in the manner in which participants use space as a proxy for thinking about time. Consistent with Experiment 1, here participants again showed a positive linear relationship between target familiarity (i.e. self-relevant episodic content) and the amount of space used to represent a given period of time. Moreover, the relationship between space and time was shown to be monotonic (i.e., more time = more space) and symmetrical across past and future events. However, in contrast to Experiment 1, there was no evidence that the impact of target familiarity diminished as a function of temporal distance (i.e., no effects of target were found when considering the slope of the regression lines). Although the influence of the differing methodologies should not be overlooked, the more proximal time-frame employed here (i.e., ±10 years) suggests that the effects of target-relevant episodic content on time-space mapping are indeed most prominent when representing more temporally tangible events.

## General Discussion

The current research revealed that the spatial representation of time varied as a function of target familiarity and temporal proximity. Across two experiments self-time was represented as occupying a greater extent of space than time relevant to other targets (i.e., best friend or unfamiliar other). Importantly, this effect was symmetrical across past and future events, and consistent regardless of whether time was mapped spatially across a mediolateral timeline (E1) or temporally along the anterioposterior plane (E2), suggesting a generality across temporal direction, mapping direction and modality.

This variation in the amount of space used to represent time is consistent with robust and systematic fluctuations in self-relevant episodic content. Put simply, a greater amount of space was used to represent temporal units that were associated with rich episodic detail than those that likely contained relatively less episodic content. As predicted, this effect held with respect to both target familiarity (i.e., more familiar = more space) and time period (i.e., more proximal times = more space), two variables that are known to influence episodic richness (e.g., [11,41–45,47,48,51–53). Further, these effects corroborate and extend extant work on the impact that psychological distance exerts on mental construal [55]–[57]. Aside from the range of effects previously reported, detail-rich simulations of proximal events (i.e., concrete representations) also seem to modulate the spatialization of time. As such, the current results speak directly to the manner in which time is cognitively represented – the very same factors that shape retrospective and prospective thought (i.e., variations in episodic content and quality) also systematically influence spatiotemporal mapping. That is, rather than time being mapped to space in a fixed, linear manner (i.e., 1 unit of time = 1 unit of space), the spatial location of temporal events is more subjectively scaled. Specifically, consistent with the predictions of construal level theory, more space is allocated to events that feature self-relevant and episodically rich (i.e., more concrete) mental representations.

A closer examination of the current findings suggests that the effect of target familiarity (i.e., more familiar = more space) was only evident when considering temporally proximal events. In particular, events from either early (e.g., 8^th^ and 9^th^ birthdays) or later (e.g., 58^th^ and 59^th^ birthdays) in life did not reveal differences in the spatial extent of one-year as a function of target (E1). In contrast, when a more constrained period of time (i.e., ±10 years) was employed, the target effect remained consistent independent of temporal distance from the present (E2). To this end, the period between ages approximately 10 and 30 years is characterized by a uniquely well-preserved level of detail of episodic content (i.e., *reminiscence bump*) [65], [66]. On the other hand, events in the more distant past or future tend to not vary substantially in terms of episodic richness, but instead are characterized by reference to typical life events (e.g., learning to ride a bicycle, having grandchildren) [67]–[69]. Thus, the contrast between the highly idiosyncratic nature of self-relevant episodic content at proximal time periods (e.g., ±10 years) and the distinctly prototypical event representations that exemplify more temporally distant episodes may underlie the current pattern of results.

While episodic richness offers a coherent explanation of the effects reported herein, it is worth noting that other factors may also be at play. For example, not only do self, best friend and a hypothetical stranger differ in terms of episodic content, but also with respect to affective significance (i.e., liking) and semantic knowledge [70]. Although it is unclear how exactly these factors relate to space-time mapping, a useful task for future research will be to establish if they influence the spatialization of time. One line of inquiry could explore spatiotemporal mappings for well known and disliked others (e.g., a despised former partner) or hypothetical strangers for whom varying amounts of semantic (or indeed episodic) information have been provided. Additionally, the complexities of space-time mapping potentially extend beyond *what* content is reactivated when contemplating past and future events. Precisely *how* an event is simulated may also impact the nature of spatiotemporal mapping. More specifically, the way an event is construed (e.g., concrete vs. abstract) and the visual perspective (e.g., field vs. observer) adopted during mental imagery have important implications for both prospective and retrospective thinking [55], [71]–[74]. Establishing whether these effects extend to the spatialization of time is also an important goal for future work.

Importantly, the present findings may have implications for understanding not only the conceptual foundations of MTT, but also other temporally-relevant cognitive phenomena (e.g., planning fallacy) [75]. If people think about self-time as being more extensive than that for others, such asymmetry may contribute to, for example, the tendency for individuals to underestimate prospective task durations for themselves, but overestimate them for others [76]. To illustrate, more activites may be seen to ‘fit in’ a given temporal duration when the amount of space ascribed to that period is enlarged (e.g., self vs. other time). Thus, by using space to understand temporal constructs, the affordances of a given period of time (e.g.,what can be achieved in that duration) may infact be derived not strictly from knowledge of the duration per se, but also from information pertaining to its spatial proxy (i.e., episodically-relevant information).

In summary, here we have demonstrated that a basic characteristic of social cognitive functioning (e.g., self vs. other differentiation) systematically shapes a decidely asocial aspect of cognition – the perception of time. Drawing from the notion that people use space as a proxy for understanding time, across two studies participants represented self-time as occupying a greater amount of space that an equivalent period related to others. Moreover, the extent of this effect reflected the quantity of episodic content typically associated with specific targets and events whereby less space was used to represent less episodically rich occurences. Establishing the behavioural implications of these findings remains an important challenge for future work.
